# Software Defined Networking for Improved Wireless Sensor Network Management: A Survey

**DOI:** 10.3390/s17051031

**Published:** 2017-05-04

**Authors:** Musa Ndiaye, Gerhard P. Hancke, Adnan M. Abu-Mahfouz

**Affiliations:** 1 Department of Electrical, Electronic and Computer Engineering, University of Pretoria, Pretoria 0028, South Africa; ghancke@ieee.org; 2 Department of Computer Science, City University of Hong Kong, Hong Kong, China; 3 CSIR Meraka Institute, Pretoria 0184, South Africa; a.abumahfouz@ieee.org

**Keywords:** wireless sensor networks, SDN-based Wireless Sensor Networks, software defined networks for sensor nodes, network management architecture, network management abstractions

## Abstract

Wireless sensor networks (WSNs) are becoming increasingly popular with the advent of the Internet of things (IoT). Various real-world applications of WSNs such as in smart grids, smart farming and smart health would require a potential deployment of thousands or maybe hundreds of thousands of sensor nodes/actuators. To ensure proper working order and network efficiency of such a network of sensor nodes, an effective WSN management system has to be integrated. However, the inherent challenges of WSNs such as sensor/actuator heterogeneity, application dependency and resource constraints have led to challenges in implementing effective traditional WSN management. This difficulty in management increases as the WSN becomes larger. Software Defined Networking (SDN) provides a promising solution in flexible management WSNs by allowing the separation of the control logic from the sensor nodes/actuators. The advantage with this SDN-based management in WSNs is that it enables centralized control of the entire WSN making it simpler to deploy network-wide management protocols and applications on demand. This paper highlights some of the recent work on traditional WSN management in brief and reviews SDN-based management techniques for WSNs in greater detail while drawing attention to the advantages that SDN brings to traditional WSN management. This paper also investigates open research challenges in coming up with mechanisms for flexible and easier SDN-based WSN configuration and management.

## 1. Introduction

Wireless sensor networks (WSNs) consist of individual nodes that interact with the environment by sensing and controlling physical parameters such as temperature, pressure and volume [1,2]. The nodes also have to interact with each other through wireless communication to achieve the sensing task [3], and are autonomous although some user-driven data collection is also possible [4]. These nodes contain computation, sensing, actuation and wireless communication functions [5,6]. Therefore, WSNs are continuously becoming important especially with the advent of Internet of Things (IoT) essential for monitoring several objects in applications such as smart cities, smart health care, smart water networks, smart power grids, smart farming and intelligent transport systems [7,8,9,10,11,12,13]. Furthermore, wireless sensor nodes are usually not tethered to a power source as they require a minimum amount of energy which is usually supplied by integrated batteries. WSNs are very flexible in their applications but also pose a research challenge due to their resource constrained and application specific architecture. With increased demand in the application of WSNs, the extent to which the technology can be applied is limited by their resource constrained nature. The main weakness of wireless sensor networks is related to the resource limitations of the sensor hardware namely processing, memory, energy and communication capabilities [14], although they are widely used due to the increased number of embedded devices available making deployment easier [15]. However, other issues associated with management of large-scale WSNs arise with the increased node deployment such as meeting the necessary Quality of Service (QoS) for satisfactory operation even as nodes scale up to very large numbers. This is an important factor to consider especially in medical and industrial applications where quality and reliability are critical [16,17,18,19].

In addition, these nodes will not only need to process data but they would also need to be flexible on variations in their tasks. Therefore, the nodes must be reprogrammable during operations when other tasks need priority. The current vendor specific sensor nodes being used in WSNs are difficult to re-task when a new parameter is required to be sensed. Hu et al. [20] give an example of 100 sensor nodes deployed in a lake WSN to measure a pollution parameter. Obviously with application specific sensor nodes being used in WSNs, task reprogramming would require each sensor to be taken out of the water and the embedded software reprogrammed in the sensor hardware. Given the need for large-scale WSNs this method would not be realistic. Some vendors have integrated Over The Air Programming (OTAP) techniques; however, the data sensing and packet forwarding protocols are still specific to the vendor. The programmer would need to study and get familiar with the particular API functions which could pose a challenge [20] and is also counter-productive as using unique programs and protocols would prevent reuse of common functionalities which if possible would expedite prototyping and development of WSNs [21].

As part of the management of a large-scale WSN, mechanisms need to be employed to allow for maintainability and system self-healing. The system should be able to change parameters based on the conditions for example reducing service quality when the energy resource becomes scarce [5]. This is a challenge with the current configuration of WSNs where the control packets and data packets are all routed through an already constrained network band. Developing a network management system for such a distributed WSN especially on a large-scale is a demanding task and is usually considered as a second phase in project planning.

The above problems are inherent to WSNs simply because each node is made to have all the functionalities from the physical layer to the application layer behaving like an autonomous system that performs both the data forwarding and network control [21]. Much as this works well especially with small-scale short range WSNs due to well-developed algorithms,it lacks simplicity and flexibility making it hard to manage when trying to implement a long range and low power WSN at a large-scale.

In order to solve this problem in management of WSNs several techniques have been researched and developed and some of these methods revolve around the concept of Software Defined Networking (SDN). SDN is a new leading architecture for networking [22,23]. It is based on the principle of dividing and separating the network into two separate planes; the control plane which determines the traffic routes and the data plane which forwards the traffic packets [24]. SDN was initially designed for traditional wired and wireless networks therefore using it for WSNs would pose a challenge due to limited node resources and other constraints inherent in the WSN architecture.

There have been some efforts to apply SDN to wireless sensor networking to enable management versatility and flexibility [21] in what are referred to as Software Defined Wireless Networks (SDWSNs). Integration of SDN would enable building of a Network Management System (NMS) to be no different from adding another application to the control plane. It would also allow for a centralized network view which is preferred by network administrators. As a result of the advantages that SDN introduces to ordinary WSNs, applications in SDN-based WSNs are being constantly developed and researched. While several management schemes for WSNs have been proposed and discussed in the past decade [25,26] and for the general SDN concept in recent years [27], there has been little emphasis on how SDN might improve management of WSNs or on the need for integration of SDN-based management architectures in WSNs. In any network that is expected to scale up to thousands of nodes and greater for use in various applications it is critical that management solutions be considered and developed [25]. There have been survey papers on general SDWSN aspects [28,29]; however, issues related to WSN management based on SDN which are crucial to the requirements of an efficient WSN are yet to be adequately addressed.

Therefore, this paper briefly reviews contributions in management of WSNs and presents a survey of the management techniques in which SDN has been implemented to improve the management of wireless sensor networks. In brief, this paper makes the following contributions.

(a) We provide an updated overview of management in WSNs as we review various contributions in network, topology, energy, security, maintenance and monitoring management of WSNs.
(b) We review SDN in general while focusing on the adoption of SDN-based management in real-world WSN applications.
(c) The general overview of the WSN management architecture based on SDN is presented and we review several contributions in the management entities of the architecture.
(d) We identify the requirements for improved and effective management of WSNs leveraging SDN thereby also highlighting available open challenges in SDN-based management for WSNs.

The approach for the rest of the paper is as follows: Section 2 presents a background on managing a WSN and Section 3 addresses general network management based on the SDN paradigm. Section 4 reviews real-world WSN applications and the benefits of SDN-based management to these applications. Next, Section 5 focuses on reviewing SDN-based management of WSNs where SDN techniques and proposed architectures for managing the network topology, energy utilization, QoS, enabling software/hardware technologies and security for WSNs based on SDN are presented. Section 6 discusses the overview for SDN-based management in WSNs. In Section 7 several open challenges in management WSNs based on SDN are identified. Finally, we conclude in Section 8.

## 2. WSN Management

Management of WSN should allow for definition of a set of functions that promote productivity and integration in an organized manner of the configuration, maintenance, operation and administration of the components and services of the WSN [25]. Several management methods have been proposed to manage functionality in the architecture of WSNs [30]. These methods take into account WSN metrics such as energy consumption, system lifespan, data latency, system tolerance to faults, accuracy in data acquisition or the Quality of Service and security.

WSN management should be simple and adhere to network dynamic behaviour, as well as provide efficiency in use of resources as proposed by Ruiz et al. [25] in the MANNA (A management architecture for wireless sensor networks) architecture. The MANNA architecture considers management policies for WSN services, functions and models by looking at management of WSNs in three dimensions defining functionality abstractions:

- WSN functionalities which include maintenance, configuration, sensor node operation (Sensing, processing, communication)
- Management levels which include application services and management of network elements (node clusters, data aggregation, network connectivity).
- Management of functional areas such as security, fault monitoring, performance and configuration.

In this paper the structure of WSN management schemes presented is based on the above protocols [30] and abstraction entities [25] however we re-categorise them according to functional relationships as follows:

(a) Network configuration management: All issues associated with network configuration and operation are categorised here. This includes protocol implementation, configuration of data acquisition, network service, network level programming issues.
(b) Topology management: This management category handles issues related to the layout of the WSN. Management of sensor node location and distribution, network activity distribution, node to node communication including gateway elements fall under topology management.
(c) QoS management: Data latency, system performance, fault tolerance, data acquisition accuracy are among the issues managed under this category to ensure an optimum WSN quality of service.
(d) Energy management: All parameters regarding energy consumption in the network are categorised here including energy sources, energy consumption minimization and system lifespan in relation to available energy resources.
(e) Security management: Considering the popularity of WSNs, more sensitive information is being transmitted over such networks thus it is necessary the network is protected from malicious attacks. This would most involve management of network security functionalities such as encryption (key distribution techniques), threat detection and recovery which are categorised here.
(f) Maintenance management: Wireless sensor network aspects related to maintaining correct operation of the network are classified under this management category. Monitoring of network performance, energy levels and faults define some of these aspects.

As shall be discussed depending on the availability of the management techniques for the categories above, WSNs can be tailored to meet at least one or more of the following design criteria:

(a) Energy Efficiency- This takes into account the ability of a system to conserve energy or allow operation on limited power for long periods resulting in improved network lifetime.
(b) Robustness- This criteria ensures that a system performs as expected regardless of varying environmental conditions or design requirements [24]. A robust system should produce desirable performance despite network variations such as node failure, power outages and instabilities resulting from node mobility. An important characteristic of a robust management system is network reconfiguration [31].
(c) Scalability- WSN nodes are expected to scale up very large numbers therefore a scalable management system should function efficiently at any network scale. Distributed management plays an important role here while reducing traffic overhead which may otherwise be all directed to a centralized activity manager.
(d) Adaptability- This criteria refers to a system’s ability to meet network variations and task demands. The system should be able to work efficiently in varying network conditions such as energy fluctuations, topology changes and task variation. The ability to reconfigure and re-task also plays an important role in meeting this criteria.

### 2.1. Network Configuration Management

Wireless sensor network configuration management integrates network configuration, performance management and maintenance management of the sensor nodes. To ensure correct network operation, the network has to be configured taking into account events and problems likely to occur in the field. Ruiz et al. [25] define some requirements for configuration management at network level such as environmental variation data, operational environment specification, topology discovery, synchronisation, node programming, node self test and node deployment. When the network is correctly configured and managed, information on the node location, administration state, usage state and energy level can easily be acquired.

### 2.2. Topology Management

Apart from MANNA, Bridge of the SenSors (BOSS) has been presented as a network management framework creating a bridge and the resource demanding UPnP(Universal Plug and Play) protocol [32]. This enables the resource constrained sensor network to deploy its services to devices based on the UPnP protocol with zero configuration. The architecture based on UPnP is composed of three parts: BOSS which forms the base node, UPnP control point and the non-UPnP nodes in the WSN. BOSS also provides the user with sensor network management services based on UPnP. It is worth noting that both MANNA and BOSS are based on the central management approach. A distributed management scheme allows for management tasks to be shared across nodes in the WSN also called Management by Delegation (MdB). This kind of setup reduces task loading on the central manager however the delegation process requires the use of intelligent nodes or mobile agents. Mobile agents are simply code sections required to distribute management tasks for execution locally on nodes while returning the resulting data to the central management node [25]. Lee at al. [31] discuss protocol functions based on MbD such as Agilla, sectoral sweepers, intelligent based power management and mobile agent based policy management.

### 2.3. QoS Management

Coupled with configuration management is performance management. The two main objectives of performance management of a WSN are the Quality of Service (QoS) and the quality of the information obtained [25]. However, there is usually a trade-off between QoS, energy consumption and network lifetime [5,25]. Depending on the environmental conditions, resource constraints, service demand; the management protocols can permit lower QoS when energy is scarce and thus extending network lifetime. Several network management protocols have been proposed in QoS management of the WSN architecture including the hybrid data dissemination framework protocol RRP, Sensor Network Management System (SNMS), Simple Network Management Protocol (SNMP) and Wireless sensor network Management System (WinMS) [31]. SNMP is a widely used management protocol that provides good management for TCP/IP based networks and is supported by several vendors in managing both wired and wireless networks. It allows management of network performance and enables fault identification. WSNManagement [33] based on SNMP protocols has been proposed as an efficient network configuration, fault and performance management system. The performance of WSNManagement showed efficiency in WSN management with a 5 petcent reduced packet loss and a 0.2 s reduced delay time while improving the lifetime of the overall sensor node system. Delay-related QoS is critical for WSNs, especially for real-time applications. Apart from WSNManagement, Zhao et al. [34] propose an optimized resource allocation solution for delay constrained Wireless Regional Area Networks (WRANs) to improve delay-related QoS management.

### 2.4. Energy Management

Energy management through software has been proposed [35] which minimises energy consumption of each sensor node by introducing a duty cycle to periodically turn on and then send the radio portion of a node to sleep. Implementing energy management schemes and energy efficient protocols maximizes the network lifetime [36]. Power management protocols providing the WSN with control over energy consumption resulting in energy efficiency have been presented severally including SenOS, Appsleep and energy level management [31]. Intelligent energy management through a prediction based dynamic method has also been proposed [37]. This technique uses a particle filter that estimates non-Gaussian processes and non-linear processes to obtain information about a target state required for optimized node sleep and sensing scheduling. This results in more energy efficiency for extended network lifetime, in addition further optimization can be performed using distributed node computing.

Similar contributions to energy management based on software duty cycling have been made by others [38,39], protocols such as Sparse Topology and Energy Management (STEM) [40] and SPAN [41,42] enable sensor nodes to go to sleep when not forwarding data. To support such energy management techniques a WSN also requires a working network management scheme in place. Lopez et al. [35] mention the need for node synchronisation when using the duty cycle method to manage energy. Works on network synchronisation have facilitated the need for node synchronisation [35]. Other energy management schemes such as energy provisioning using batteries and energy harvesting have been been presented in the past years [43]. Algorithms are being developed to further enhance the energy harvest method resulting in network self-sustainability and one such algorithm is the Energy Aware Adaptive Sampling Algorithm (EASA) proposed by Srbinovski et al. [44]. This energy management technique is suitable for power hungry nodes with the capacity to harvest energy and given the required power to process the algorithm, it can be implemented on any WSN.

### 2.5. Security Management

Management of security is difficult to provide as WSNs are mainly made up of ad-hoc wireless networks with intermittent connectivity and resource limitations [25,45]. This leads to vulnerability to threats that could be internal, external, malicious or even accidental. Data and resources can thus be stolen or modified and a denial of service attack is also possible. Security management for example has been approached through the use of key management schemes [26]. Reegan et al. [26] mention various requirements in managing security such as authentication, confidentiality, integrity, availability and authorisation. A network with high security features should be developed to meet these requirements however, the challenge here is the limited resource in bandwidth and energy inherent to WSNs [46,47]. Management of security therefore has to be based on effective key distribution [26] taking into account features such as bandwidth, sensor memory, transmission range and the necessary know-how [48]. Several key management schemes have also been discussed by Reegan et al. [26]. Much more recently Key management based on the dynamic clustering and optimal routing choice of the Mobile Sink has been proposed [49]. The scheme extends static key management to dynamic key management with an improved flexibility while satisfying storage efficiency and connectivity. A further contribution has been made by proposing a hybrid key management scheme which takes advantage of both the Polynomial Pool and Basic Random (PPBR) key pre-distribution techniques to improve the difficulty in cracking the key system [50]. To tackle issues related with link connectivity in key management, the tree base path key establishment method is used.

### 2.6. Maintenance Management

Maintenance management as part of network management would enable higher QoS and an extended network lifetime. Faults in WSN occur frequently due to interruptions in connectivity, energy shortages, changes in the environmental conditions and other network events expected and unexpected. The network therefore, should be configured, managed and maintained in a manner that allows for self-healing and fault tolerance resulting in a robust characteristic enabling the WSN to provide useful information even when some nodes fall out of the network. Dayal and Kumar [51] have presented various fault mitigation techniques through management of the network topology.

An important aspect of maintenance management and fault mitigation is network monitoring required to collect information on the state of the network (e.g., node energy levels, bandwidth and link states). Thus, interfaces and tools have been developed to provide a visual representation of the network state such as Mote-view [52], SNAMP [53], spy-glass [54], tinyviz [55], nanomon [56] among others. Lee et al. [31] mention that systems such as the mote-view and others that have a central management scheme with all analysis data being sent collectively to a central server have the disadvantage of being passive and lack flexibility in configuration in an event of fault occurrence. This can be seen as a leverage for SDN in solving this lack of flexibility, an aspect that becomes visible as management of WSNs based on SDN is explored in this paper. An Energy-efficient Passive MOnitoring SysTem (EPMOSt) has been proposed taking into account the need for energy efficiency in WSNs [57]. Similar to WSNManagement [33] EPMOSt uses an SNMP agent to provide monitoring information with reduced energy consumption. This energy efficiency ensures longer network monitoring lifetime.

### 2.7. Comparison of WSN Management Systems

Notable WSN management systems discussed above are summarised in Table 1 and evaluated against the WSN design criteria. From the table we see that BOSS, Mobile Agent Based Power Management, WinMS, AppSleep and EpMOSt are promising management schemes meeting atleast three of the criteria required for an efficient management system with WinMs being most efficient.

## 3. Network Management Based on SDN

**Figure 1:**
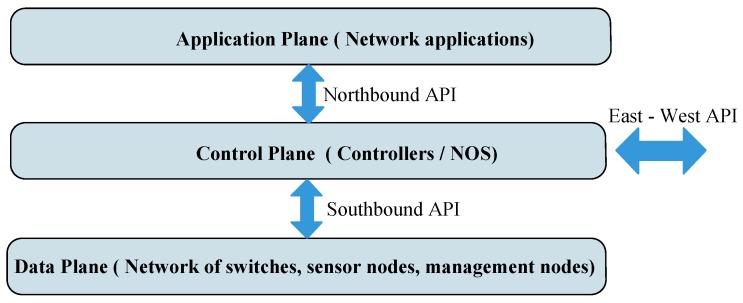
SDN overall architecture.

The principle concept behind Software Defined Networking (SDN) is based on the separation of the control plane from the data plane in the architecture of WSNs. This new paradigm allows for a logically centralized controller which is a central program acting as the Network Operating System (NOS) thus controlling and managing the overall behaviour of the network. The network devices such as sensor nodes and switches exist in the data plane and simply forward data based on flow instructions provided by the controller. This approach to network management allows network configuration to be performed globally as opposed to a distributed management scheme which may require individual configuration of network devices to change network behaviour. The overall basic architecture is shown in Figure 1.

The SDN architecture integrates Application Programming Interfaces (APIs) which provide a working interface between the application, control and data planes. Between the application plane and the control plane are the northbound APIs and between the controller and the data plane exist the southbound APIs. The Southbound APIs facilitate for the flow of control traffic from the controller to the devices in the data plane. OpenFlow [20,21,58] is standard commonly used as a southbound interface [27] and most vendors are producing more OpenFlow capable network infrastructure. East-West APIs enable controllers that are in the same domain or neighbouring domains to communicate with each other [59]. It is important to mention here that SDN is not about improving the performance of the network but rather SDN has been seen to have the potential to simplify network management and allow for innovation through network programmability. Updates to network control and management are made as easy as installing new applications on a computer [60]. SDN changes the network administration problem into a programming problem increasing flexibility, other potential benefits of SDN in WSNs [14] include:

(a) Energy management: WSN nodes are energy constrained and thus need energy-efficient protocols to be employed in sensor networks. SDN can provide an energy efficient way for network management of WSNs. This is possible as having a logically centralized control plane maintains a complete view of the entire WSN and thus can reduce the power consumed by nodes in maintaining that view locally. Control plane functions can manage all the routing protocols saving nodes from following application specific protocols which might drain energy depending on traffic. Alternative energy management techniques can also be employed at the control plane.
(b) Configuration management: The complexity of network management can be simplified with due to the increased flexibility of SDN, new routing protocols can be employed easily without reconfiguring the nodes and also it reduces the need for compiling different versions for the same network applications for different sensor nodes. Thus, if a new management and control method becomes available, it can easily be deployed resulting in efficiency of operation.

Network management with different vendor specific network hardware can be challenging [27]. Network operators need to adjust to low-level vendor specific hardware configurations to implement complex high-level network management policies. Network management schemes such as those discussed in the previous section encounter difficulties in changing the policies embedded in the underlying network infrastructure. The direct result is the existence of application specific networks with few possibilities for improvement and innovation. New possibilities in management of a network and configuration can be introduced by SDN. SDN helps solve some of the problems of network management by enabling easy and frequent changes to the network function and state, allowing configuration of the network in high-level languages and providing visibility and control over the entire network making it easier to troubleshoot problems in the network.

In traditional wired/wireless networks, network operators have to implement and configure networks using a set of low-level device commands in the command line interface [27]. This is especially a problem with constantly varying network conditions. Operators have to manually adjust configurations frequently to meet these changes leaving room for misconfiguration and mistakes in implementing changes [61]. With SDN it is much easier to integrate new protocols and ideas into the network through the use of a centralized controller. SDN introduces a global approach to management of network configuration rather than local management which would require configuration for each of the network devices to implement changes or integrate new services. Several work has been researched and proposed on the potential benefits of implementing SDN in both wired and wireless networks [14,21,24,28,62]. Kim and Feamster [27] have designed and implemented Procera which is an event driven control framework for a network based on SDN. Procera focuses on network management by allowing operators to implement high-level policies which are translated into a set of forwarding rules. These rules are then used to enforce policies on the network hardware using OpenFlow [20,21,58,63]. This management process takes place at the policy layer of SDN which exits as part of the northbound API. There have been numerous prior researches on applying SDN in management of computer networks leaving room for study on how SDN can facilitate and enhance management of IoT. An SDN-based management architecture for IoT with a focus on Machine to Machine (M2M) infrastructure has been proposed by Huang et al. [64]. The proposed framework consists of a centralized controller, M2M nodes, a gateway linking non-M2M protocol general nodes to the controller. The framework was tested successfully for routing reconfiguration without the need to deploy new infrastructure.

## 4. WSN Applications and the Need for SDN-Based Management

Wireless sensor networks are able to integrate various sensing capabilities thus providing support for various real-world applications. This flexibility is accompanied by several research challenges in providing effective management for the application considering the resource constrained nature of WSNs. The various benefits that SDN introduces to these management challenges allows it to be utilized in several applications such as in environmental applications, health care, military and in home networks. We shall briefly discuss these application scenarios and bring to light the benefits of SDN-based management in these areas.

### 4.1. Environmental Applications

A popular environmental application of WSNs is based on the measurement of physical parameters such as temperature, vibration, sound, chemicals and gases. This has led to application in scenarios such as the agriculture industry for irrigation or animal monitoring, water industry for development of smart water grids to enable efficient distribution and control of potable water [11] and in disaster relief applications for monitoring and fighting forest fires and also in earthquake detection and rescue operations. Other applications include environmental control of pollutants and marine monitoring requiring long term unmanned WSN operations. However, such applications may require deployment of sensors over very large areas, harsh environments or even hard to reach regions which may result in dependence on sensor energy supply which affects network lifetime and the ability to alternate tasks upon change of sensing requirement or upon node failure [65]. There is therefore a need for effective topology, energy and maintenance management of which integration of SDN introduces to the application scenarios. SDN-based management would allow for a centralized network view of essential parameters suitable for large-scale applications, where energy is crucial SDN techniques provide effective resource allocation through software duty-cycling and by enabling easier use of traffic engineering, congestion control, load balancing [66] and mobility management. SDN also provides an effective interface for sensor re-programmability necessary for function alternation without requiring additional hardware such as FPGAs [9].

### 4.2. Medicine and Health Care

WSN application in heath care has been considered to have the potential of being beneficial to the quality of health especially for the chronically ill and elderly patients. WSNs can also be used for tracking of patients and doctors in hospitals which can prove useful in saving lives. A controversial application of WSNs in health care is the use of implantable sensor systems to monitor patient activities resulting in Wireless body Area Networks (WBANs). Darwish et al. [67] identify challenges to implementing WBANs among which are sensor maintenance once body-worn, energy management for sensor batteries considering that charging would be pose a challenge especially for the elderly as they have to remember to charge multiple sensors, insufficient bandwidth resource to allow for transmission of large amounts of medical diagnostic data, meeting the QoS requirements of health monitoring hindered by WSN resource constraints, reliability of critical medical data packets being delivered, security and privacy of medical data, scalability and sensor mobility challenges as patients move about in their daily life. SDN-based management would provide a solution to several of these highlighted challenges based on the ability to provide energy and maintenance management from a central location which could be a task of hospital administration for instance. Furthermore, SDN techniques are being developed to reduce traffic overhead data to mitigate bandwidth problems [68,69] and also to ensure accuracy of data and fault monitoring more efficiently. Considering the possibility of implanting multiple body sensors in close proximity which may result in interference affecting the QoS and application performance, SDN provides a solution based on separation of destructive interfering nodes. Security and privacy is a key issue in health applications and SDN provides several encryption algorithms that can be delivered on demand, the encryption techniques available are discussed later in this paper.

### 4.3. Military Applications

In military applications WSNs can be used to detect presence of enemy or friendly forces, assessment of terrain and chemical sensing of weapons. Recently Fraga-Lamas et al. [70] reviewed the possibility of applying IoT to warfare to increase efficiency. Uses of WSNs for defence IoT highlighted included application in fire control, inventory tracking, fleet monitoring, energy management and management of the health and safety of troops. However, to be suitable for use in defense and security WSNs need to meet operational requirements such as robustness, ease of deployment, interoperability/adaptability and most importantly security. SDN can be leveraged to better achieve these requirements as it maintains a global control of the network infrastructure in the field which better manages system security and issuance of new task protocols during operation. SDN-based management also provides an opportunity to easier deploy mobility algorithms to handle constant movement of troops as these algorithms can be configured and managed in a more resource capable global controller.

### 4.4. Wireless Home Networks

WSNs for home use have seen increasing demands with arising smart homes and buildings in general. Homes are now composed of a network of light, motion and temperature sensor nodes that can be integrated in appliances to introduce smart capabilities in a home. This network can also be linked to the Internet to allow users to remotely control these home appliances. However, in addition to a network of smart devices is a network of other communication technologies such as WiFi, ethanet, cellular and power-line communications. The challenge with such a heterogeneous network is the lack of an automated system to enable network optimization by selecting the best communication technology to use, this decision is usually made by the user manually. Soetens et al. [71] have proposed an SDN-based management technique to improve management of such heterogeneous home networks by using OpenFlow-enabled link switches. SDN techniques have also been proposed to enhance the development and management of smart homes [72]. Smart device use is increasing in homes and this creates difficulty in management especially with the advent of video streaming services such as Youtube and Netflix. User demand and control becomes an important management factor leading to the need for smart home network management based on SDN. In addition, these user applications such as voice, video, gaming and others have to compete for bandwidth resulting in poor quality of service. Kumar et al. [73] have proposed and experimented on an SDN-based solution that allows users some control over the service quality for their devices enabled by ISPs. Other bandwidth allocation techniques for managing home applications like video and voice have been proposed based on SDN to improve quality of service including home slice [74], Network Assisted Video Streaming (NAVS) [75] and SDN@home [76].

## 5. Management of WSNs Based on SDN

**Figure 2:**
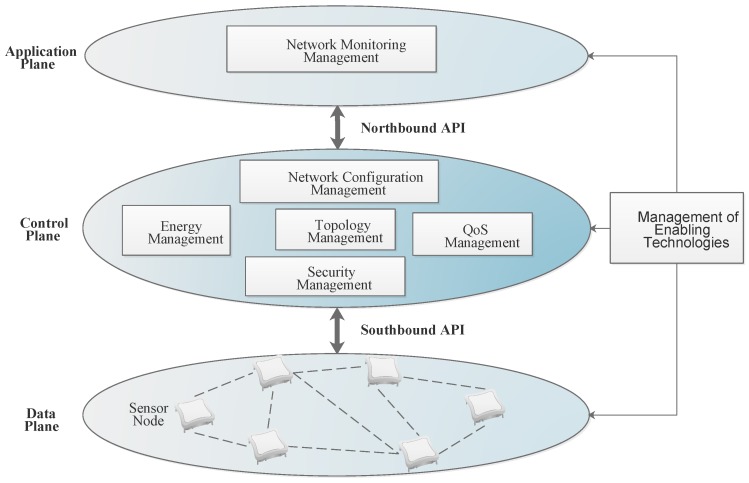
An SDN-based wireless sensor network management architecture, adapted from [28] (Republished with permission of IEEE, from Wireless Software Defined Networking: a Survey and Taxonomy, I. T. Haque and N. Abu-Ghazaleh,18, 2731–2737, 2017 Copyright).

WSNs are composed of a network of sensors that are deployed to measure parameters such as temperature, pressure and air quality. The sensor network may exist as hundreds or even thousands of resource constrained nodes. Also taking into account the inherent challenges of WSNs in terms of energy requirement which is directly linked to network lifetime, the application specific nature with vertical integration [27] where manufacturers and vendors control the end products of the devices as well as the software and hardware of their infrastructure . Another challenge introduced as the nodes are deployed in the field is the management of the network topology [77] where we have to consider the coverage of the network and Quality of Service in an environment with varying conditions at a limited energy supply. Integration of SDN in WSN has been proposed [14,21,24] to mitigate these challenges. Integration of SDN would require consideration of how the network will be managed to ensure efficient performance and resource allocation. The WSN architecture based on SDN can be divided into managerial entities similar to the management functionality abstractions as mentioned by Ruiz et al. [25]. Figure 2 shows this architecture [28] adapted with a further focus on the management entities we will consider in our review and evaluation of SDN-based management for WSNs. The architecture shown depicts an SDN-based WSN with a centralized controller thus providing a global view of the entire network resulting in efficient management. Management of the network topology, energy, configuration, QoS and security are done centrally in the control plane while management of enabling technologies can be implemented at all levels of the architecture. In the data plane enabling hardware such as SDN-enabled sensor nodes with multiple sensing capabilities and hardware capabilities can be installed while programming of the nodes for various applications can be done and managed centrally in the control plane.

### 5.1. Network Configuration Management

Network configuration management is a functional area that will allow the sensor network to meet its required specification. In an SDN-based WSN architecture, the first challenge with meeting the requirements has to do with SDN support for the WSN architecture [28]. SDN was designed for conventional networks which are different from WSNs and therefore it is necessary to configure the network to enable SDN support efficiently. This is particularly important in harnessing existing network management protocols and architectures which can provide the SDN-based WSN with a proper network management model [29]. Several proposals have been made towards configuring and extending SDN functionality for wireless sensor applications. Sensor OpenFlow (SOF) [20,21] is one such proposal which serves as a standard communication protocol between the data plane and control plane and is a core component of WSNs based on SDN. The idea is based on the data plane that is composed of sensors performing flow-based packet forwarding and the control plane consisting of a controller handling all the network intelligence at a central place (Centralized Control). This allows the data plane to be fully programmable by customizing the flow table of each sensor. The challenge with this concept is that it is built on the fundamental OpenFlow assumption that the underlying network is made of Ethanet switches and IP routers which are wired protocols useful in cellular and enterprise backbones and data centers; and not so much in WSNs. Moreover, using the centralized controller would raise questions on the management of security in an event of attacks directed towards the controller [14]. Thus, adequate network security management has to be in place.

Software Defined Wireless Network (SDWN) is yet another solution that has been prototyped to extend SDN functionality in wireless and mobile communications specifically providing support for SDN in Low Rate Wireless Personal Area Networks (LR-WPANs) [60]. SDWN uses energy management techniques such as duty cycling, in-network data aggregation and cross layer optimization to reduce energy usage. However, adapting the SDWN architecture to integrate and configure SDN for WSNs to enable efficient energy use needs further investigation as the SDWN requires messages to be passed between control and data layers which may strain the already resource constrained WSN.

Galluccio et al. [78] have proposed SDN-WISE (SDN-WIreless SEnsor network), a technique which makes sensor nodes programmable as finite state machines so as to enable them to run applications that cannot be supported by stateless solutions. It provides an API which allows developers to program the SDN controller in a language of their choice enabling simplicity and flexibility in the management of network programming. This is especially useful when managing a large-scale and long range wireless sensor network.

Configuration of SDN-based WSNs should allow the SDN protocols which are address-centric to address the inherently data-centric WSN. A solution [21] to this would be integrating the use of IP stacks such as uIP, uIPv6 [79,80] based on Contiki OS [81], Berkley Low power IP (BLIP) based on Tiny OS [82,83] for low power networks. As a result of this integration IPv6 based low-power wireless Personal Area Networks (6LowPAN) have been realised. Management architectures based on the 6LowPAN protocol have thus been proposed including the LowPAN Network Management Protocol (LNMP) [84], 6LowPAN-SNMP [85] which is an extension of the existing SNMP [31] protocol and a further network management architecture based on SNMP for 6LowPANs proposed by Feng et al. [86]. These management architectures although used for various wireless networks provide a model for developing a proper network management architecture for the WSNs based on SDN [29].

Another aspect of network configuration includes management of the flow of control and data traffic which use the same network path (in band) in WSNs unlike in wired SDN applications where a dedicated control channel exists for communication between the controller and the data plane. This would pose a challenge for WSN control data if not managed due to variations in connectivity from link and node failures, mobility, routing and other similar network parameter variations. In line with this, proposals have been made to reduce control traffic as a management solution, Luo et al. [21] for instance propose a method of only sending a single packet meant for a flow setup request to the controller when a table mismatch occurs and suppressing the proceeding packet requests with the associated packet having the same destination address as the first packet until a corresponding request is received from the controller or when a set expiry time is reached.

### 5.2. Topology Management

WSNs are different from the conventional networks that SDN was initially designed for. WSNs have limited resources and use the same channel for node to node communication and data processing (in-network processing). SDN allows for decoupling of control plane and data plane however, for WSNs the control and data packets will still use the already resource constrained and dynamic WSN topology. In management of the network topology, the technique employed should be able to support node mobility which results in topology changes including dealing with unreliable wireless links. These functions should be in tandem with management of QoS which ensures that the network is robust. WSN topology management based on SDN can be divided into management of the following:

#### 5.2.1. Scalability and Localization Management

The architectures reviewed so far are based on a centralized controller in a flat topology which is easy to deploy and manage. Gante et al. [14] for instance introduce smart management of SDWSNs to improve efficiency and overcome inherent difficulties of ordinary WSNs. The management scheme is based on a proposed base station architecture for WSNs with an integrated controller. The controller creates forwarding rules that are placed in flow tables from location data obtained through localization techniques processed in the application layer of the architecture. However, flat topologies like this are limited to short range and small scale applications and as networks scale up to very large numbers of nodes there is need to integrate topologies that are hierarchical or provide a virtual overlay of the physical network [87] to support efficient scalability and localization. A few architectures are available for network management designed towards improving SDN-based WSN management efficiency by using distributed multiple controllers rather than a single centralized controller. This also enables efficient scalability and a reduction in overhead control data as it is not necessary to communicate all control data centrally. Distributed controllers also allow for effective security and QoS management as the WSN is not solely dependent on one controller, making the system more reliable during attacks or failures in the link to the central controller.

**Figure 3:**
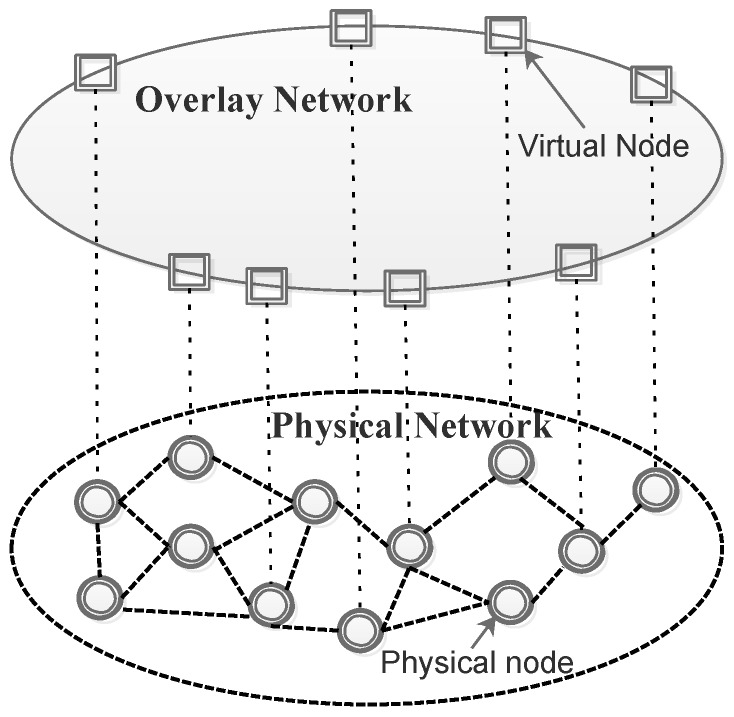
Topology based on virtual overlay [87] (Republished with permission of IEEE, from SDN-Based Application Framework for Wireless Sensor and Actor Networks, Zhou, J.; Jiang, H.; Wu, J.; Wu, L.; Zhu, C.; Li, W., 4, 1583–1594, 2017 Copyright).

A hierarchical architecture called Software Defined Clustered Sensor Networks (SDCSN) has been proposed [88] to use multiple base stations as controllers that also play the role of cluster heads. A large-scale group of nodes is divided into clusters and there is a cluster head for each. The cluster head controls and coordinates the sensor nodes in each cluster and all the information processed in each cluster is routed to the cluster head. Management of such an architecture requires enabling the sensor nodes to function as controllers effectively enabling multiple controllers in the network. Oliveira et al. [69] design and implement an architecture based on multiple controllers within a WSN in a framework called TinySDN based on Tiny-OS with a design structure that consists of SDN-enabled sensor nodes and an SDN controller node. This addresses issues such as in-band control, higher communication latency, and limited energy supply. The downside to this management approach is that the cluster head can also become vulnerable to attack posing a network security risk [87] however self-stabilisation techniques for re-selecting a new cluster head upon such an event have been proposed [89]. To manage the network with more flexibility for integration of various functions an architecture based on network virtualisation has been proposed [59,87,90]. Basically, a virtual overlay topology can be built to certain specifications based on an actual physical WSN based on SDN [87] as shown in Figure 3.

Another hierarchical architecture proposed to manage scalability is context based logical clustering [91,92]. This is based on logically clustering sensors according to context (gathered data type) regardless of their physical distribution based on HyperFlow [93] unlike other clustering methods proposed which allow clustering of adjacent nodes. Clustering sensors according to context would allow for data and resource sharing regardless of network expansion. Each cluster has a local controller which maybe physically distributed but logically synchronised hence referred to as logical sink in the virtual network overlay. The performance study shows that this technique improves network stability effectively [92].

Sensor data without location information is not useful especially in large-scale applications with hundreds to thousands of nodes. SDN in WSNs provides the possibility of obtaining location information with a good level of accuracy by implementing proposed localization algorithms [94,95,96,97,98,99]. The localization algorithm to be implemented and managed can either be centralized or distributed depending on the mapping information and level of accuracy provided by the algorithm. More recently a localization node selection algorithm based on the SDN and the Cramer-Rao Lower Bound (CRLB) metric has been investigated and proposed [100]. The algorithm makes use of the global network view that the SDN controller has while satisfying the need for energy conservation and the simulation results presented a significant improvement in the localization performance.

#### 5.2.2. Mobility Management

Sensor nodes in an SDN-based WSN are susceptible to movement and this can cause variation in packet transmission and execution of tasks making it necessary to monitor and manage the movement of nodes in the network. The challenges to consider when integrating a mobility management scheme includes handling the effect of nodes entering the network and the nodes leaving the network on QoS, execution of functions and other network attributes. A few solutions and processing steps that the SDN controller can provide to prevent problems associated with nodes entering and leaving the WSN have been outlined by Zhou et al. [87]. Considering IP support for nodes in SDN-based WSNs [79,82] research has been conducted on mobility management in IP based WSNs based on TinyOS implemented and tested on a 6LoWPAN [101]. A mechanism based on IEEE 802.15.4 [102] standard has been investigated and implemented to manage mobility in a distributed network architecture with promising implementation capacity in a real system [103]. There has been several contributions on mobility management in Wireless Cellular Networks (WCNs) based on SDN [104,105,106,107], whether or not these management mechanisms can be applied and integrated in SDN-based WSNs is a worthwhile research area.

#### 5.2.3. Communication Management

**Figure 4:**
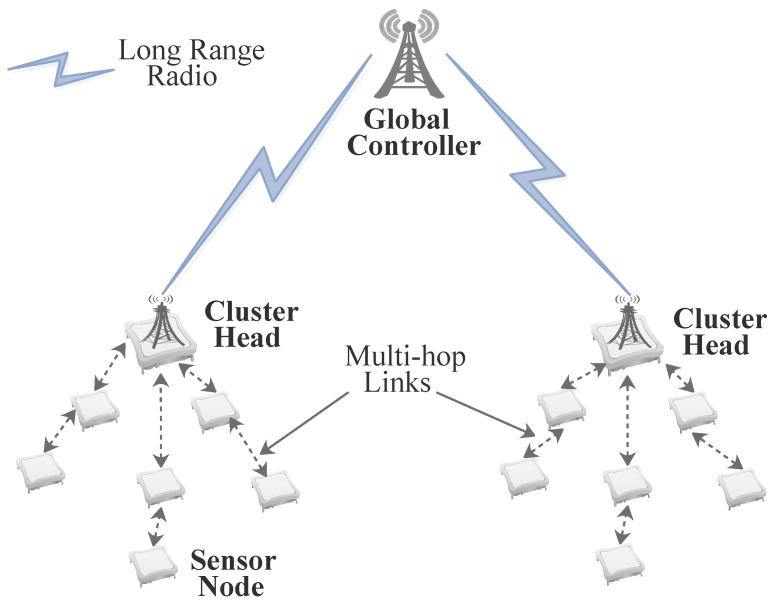
Hybrid radio management.

Integrating SDN in the WSN topology enables management and operation of a network with sensors of different architectures and manufacturers with little or no challenge with compatibility issues. These nodes can be densely located with neighbouring sensor nodes appearing very close to each other and hence the need for an efficient communication scheme to be deployed. The communication scheme should take into account energy consumption and signal quality however, the primary focus should be on conservation of energy to improve network lifetime. Multi-hop techniques based on short range radio communication such Meshlogic and ZigBee [46] have been proposed [108] for dense WSNs enabling consumption of less energy compared to direct communication. In SDN applications employing clustered hierarchical topologies, long range radio can be used for node to cluster head communication. This would allow for continuity in network operation even when a few nodes in a cluster fall out due to the direct communication between the cluster head (SDN local controller) and each node. The proposed radio technology to be implemented in such scenarios should also require less power such as the LoRa technology intended for Low Power Wide Area Networks (LPWAN) [109,110]. A hybrid of multi-hop techniques and long range radio can be used to manage a large low power WSN based on SDN where multi-hop communication is implemented between nodes in a cluster and long range radio is used between cluster heads and the global controller as shown in Figure 4.

#### 5.2.4. Network Monitoring

To improve manageability and ease of implementing a WSN based on SDN, network monitoring is essential as it enables the display of all necessary sensor information such as details of the sensor node hardware, software, and battery level at the management station [111]. In SDN, network monitoring applications have been developed based on the Openflow capabability to provide a mechanism for collection of flow level information and statistics. These applications through the controller are able to query nodes from time to time. The accuracy of the monitored information is dependent on the frequency of these queries. Techniques such as Payless [112] have been proposed to improve the efficiency of this query process on monitoring accuracy and network overhead. Other proposed network monitoring tools based on this concept for traditional software defined IP networks include OpenSketch [113], OpenTM [114], FlowSense [115] and OpenNetMon [116]. There has been little effort in investigating these techniques or new techniques for monitoring SDN-based WSNs and is still an open research area. Recently however, work has been presented on a network measurement architecture based on SDN for monitoring of WSN information such as routing path per-packet, the loss ratio for each link and the delay resulting from each hop in a multi-hop configuration. TinySDM [117] a software defined measurement architecture built on the TinyOS [83] platform enables easy customisation and execution of various measurement metrics and allows for quick and efficient implementation of measurement tasks.

#### 5.2.5. SDN-Based Network and Topology Management Classification for WSNs

Classification criteria is an important factor to consider when integrating a management system into a network. Choice of the management system would depend on the management function, controller configuration to which the architecture is suited and control traffic channel used. Table 2 summarises and compares the architectures available in network and topology management of WSNs based on SDN against classification criteria.

### 5.3. Energy Management

Energy management focuses on the several techniques available to leverage SDN in ensuring that energy utilization is minimized in an already energy resource constrained WSN. Several proposals for adopting energy management in the SDN controller for the global balance of energy utilized in the WSN have been made. Integration SDN in WSNs allows for energy consuming tasks such as data traffic routing decisions to be handled in the controller leaving the energy constrained sensor only with the task of forwarding data traffic inherently reducing the amount of energy used. Part of the topology management of WSNs based on SDN is focused on using SDN for resource allocation control. To enhance resource allocation architectures and topologies likewise have been proposed such as the SDWN [60] and Smart [14] both executing controller functions on the sink node freeing remaining sensor nodes from functions like localization, QoS and mobility management resulting in energy efficient operation. Similarly, a general framework for has been been proposed that places the controller at the base station with centre nodes in each cluster integrating switching and communication functions with the controller based on OpenFlow [119].

Management of energy has also been demonstrated by establishing control through software in a method referred to as duty cycling, focusing on reducing the amount of energy consumed by the sensor nodes through control of the sleep and wake up states. This technique has been proposed severally for managing WSNs [35,120,121] however SDN-based architectures rely on cross layer communication between the decoupled control and data planes which may require a robust link for the control traffic flow to be optimized in addition to the need for energy saving through data aggregation; using techniques that switch nodes on and off may result in unreliability in the communication links. Multi-tasking of WSNs has been proposed as an architecture based on SDN to minimize energy utilization in [122]. Multi-tasking is possible through the ability of the sensor node to carry out multiple tasks at the same time achieving different sensing outputs and to achieve a desired Quality of Sensing (QoSen) a task is carried out collectively by multiple nodes. Zeng et al. [122] present an architecture allowing for energy efficiency in sensor scheduling and management while producing the required QoSen. A Mixed Integer Linear Program (MILP) is formulated to take into account three objectives: sensor activation, task mapping and sensing scheduling. Further work is also done on developing an on-line algorithm to handle the dynamics associated with nodes entering the network (mobility) or leaving the network (mobility or node failure) with simulation results showing equal energy efficiency as a global optimized network with the advantage of having a lower rescheduling time and overhead control data.

Inherent in the SDN-based management is a high control traffic overhead to maintain the global view of the logically centralized controller; a process that requires more energy for operation and thus affecting the energy efficiency of the network. Huang et al. [123] propose a prototype based on reinforcement learning to improve energy efficiency of SDN-based WSNs. The technique reduces overhead control traffic by filtering redundancy and performing a load-balancing routing mechanism in accordance to data flow distribution with the required QoS. Experiments on the proposed SDN-based WSN prototype showed an improved energy efficiency performance.

Energy management techniques such as data aggregation and information fusion [124] coupled with multi-hop communication used in WSNs [125] can be extended and applied to within each cluster. Multi-hop links can take advantage of the densely located sensor nodes to transmit data at low power [108]. More recently, an efficient energy harvesting management technique based on wireless power transfer for SDN-based WSNs has been proposed [126]. The mechanism uses dedicated wireless power transmitters to transmit energy to sensor nodes in the network thus increasing the network lifetime. A utility function is defined to manage the energy distribution and the maximum energy required to charge the nodes to enable maximum optimization while meeting the minimum energy required by each sensor node. Ejaz et al. [126] also propose an accompanying energy efficient scheduling system for the energy transmitters during each charge process. Table 3 compares the energy management schemes available for SDN-based WSNs.

### 5.4. QoS Management

The Quality of Service management covers all aspects of ensuring an acceptable delivery of service in a network. This includes management of system tolerance and reliability, packet latency, event detection, robustness and maintenance. The SDN architecture allows for the QoS management tasks to take place in the controller by virtue of being logically centralized. The SDN-based WSN environment varies due to factors such as topology changes, node failure, bandwidth availability and energy fluctuations therefore, there is need to integrate a mechanism for reliability of the network. Lu et al. [127] study the reliability of the WSN architecture based on SDN through the use of models based on continuous time Markov chain and continuous stochastic logic techniques. The results show that network reliability is affected by the number of controllers and sensors and their respective failure rates. From the results a proposal to integrate more than one reliable controller as a reliability management strategy is made.

Management of reliability has been classified into the reliability of communication systems and reliability of tasks [87]. Management of communication reliability can done through methods such as providing back up nodes and redundant links [87]. Function alternation has been proposed to manage reliability of sensor tasks by enabling neighbouring sensor nodes to take over damaged sensor node tasks automatically providing a self healing mechanism [65]. The SDN controller can also provide a back-up for sensing tasks and information to improve the reliability of tasks [87]. The back-up information can be used for re-tasking neighbouring sensor nodes to take up the task of damaged or unreliable nodes in SDN-based WSNs. The SDN OpenFlow concept has been demonstrated and exploited to address issues of performance reliability in WSNs [128]. The idea presented is based on the inherent characteristic of OpenFlow to control and monitor sensor traffic enabling a reliable routing and load balancing mechanism. In ordinary WSNs the reliability of multi-hop and end to end communications is still an unsolved and difficult issue to manage and hence Flow-sensor has been proposed to produce better reliability as compared to typical sensors by generating a lower amount of packets reducing the traffic overhead in constrained networks [128]. Another QoS parameter is robustness which is a characteristic that defines a system’s ability to meet its expected performance [24] under unreliable environmental conditions. To manage system robustness it is necessary to consider factors affecting and interfering with the system and monitor the interference patterns in a proposed statistical machine learning technique [24]. This technique would allow for the prevention of possible interference based on previous behavioural patterns of interference. In managing latency TinySDN [69,118] has been proposed however, there is need for further research on managing latency and event detection.

### 5.5. Security Management

The architecture of WSNs based on SDN is such that a logically centralized controller poses a security risk as any compromise in the controller, cluster-head in clustered topologies or even the link to the controller can affect the system QoS. A malicious attacker can introduce false data and parameters in the network or a Denial of Service resulting in system unreliability. A few contributions have been made in managing security for SDN-based WSNs, Flauzac et al. [129] for example describe the need for security management in both wired and wireless networks based on SDN for Internet of Things (IoT) and propose a security model for IoT SDN architecture. An extension of the proposed architecture for IoT is made to include sensors integrating the aspect of guaranteeing the security of the network based on the grid of security concept embedded in each controller. The grid of security concept has been presented previously as an approach to securing networks by ensuring that communication takes place between trustworthy devices regardless of system policy control supported by a communication model to help structure the service distribution of security among nodes [130]. The overall concept is to decentralize the management of security of the network to ensure continuity in security during failure.

Another security management contribution has been the integration of Secure Multiparty Computation (SMC) to secure private sensory data in SDN-based WSNs [131]. The focus is the application of SMC in securely processing sensory data between the SDN-based WSN and the web server. Sun et al. [131] present a security model based on the SMC protocol allowing clients in the SDN-based WSN to connect and disconnect to the web server arbitrarily. A lottery SMC protocol is constructed for the performing selection in SDN-based WSNs with an encryption based on the layered homophobic function.

### 5.6. Management of Enabling Technologies

#### 5.6.1. Enabling Software

A major part of WSNs based on SDN is software, which enables the management and control of sensor nodes and also forms the basis for sensor operations. The SDN-based architecture allows for the management and control of enabling software taking place at the application, control and data planes in a particular manner that enables application programmers to adjust the sensor functionalities by invoking different programs making the sensor node behave like a tiny computer. This also calls for the sensor nodes in the data plane to have an operating system however, a less complex one compared to personal computers because sensor nodes have limited processing power and memory [62]. There are several sensor operating systems that enable the integration of sensors into the architecture of an SDN-based WSN. Choice of operating system affects the memory management and protection of sensor nodes with memory management being classified into either static or dynamic memory management. Static memory management is fixed and simple hence useful for constrained memory nodes but is not flexible due to the unavailability of allocating memory at run-time. Dynamic memory management on the other hand is flexible as it provides for allocation and de-allocation of memory at run-time [132]. SDN-based WSNs provide for multiple execution of functions thus the need for memory protection to prevent the mix up of process address spaces. TinyOS [83,133] written in nesC has been structured for low-power wireless sensors and embedded systems that have limited resources. TinyOS provides for static memory management with memory protection. The use of TinyOS in WSNs based on SDN has been demonstrated in [69,101,117]. Contiki [81,134] has been presented as a lightweight operating system with support for dynamic network loading and redefinition of node programs. Feasibility of dynamic loading and offloading of programs in a resource constrained network is demonstrated [81] based on Contiki. Contiki supports dynamic memory management and memory block functions but does not provide a management mechanism for memory protection between function executions [132]. Other available operating systems include MultimodAl system for NeTworks of In situ wireless Sensors (MANTIS), Nano-RK and LiteOS [132] however there is little or no implementation of these operating systems in SDN-based management of WSNs but rather for ordinary WSNs. Table 4 compares the available sensor node operating systems for integration in SDN-based management of WSNs.

An important feature of SDN in WSNs is to provide a global view of sensor nodes allowing for efficient program delivery when forwarding control data or updating node software for a new task. The architecture hence allows for this program delivery through Over The Air Programming (OTAP) Techniques which contribute to the efficient management of enabling software. Use of OTAP in downloading of roles to nodes via a multi-hop wireless link in a reconfigurable SDWSN has been demonstrated in [136]. Efficiency in OTAP has been further demonstrated by the use of SDN-WISE, an SDN solution for WSNs. A hands-on demonstration has been done using SDN-WISE to reprogramme WSNs allowing for various logical sensor networks to exist together while using the same node hardware [137].

#### 5.6.2. Enabling Hardware

Enabling hardware forms part of the infrastructure of WSNs based on SDN and an important component for managing and enabling the SDN integration into WSNs is the SDN-enabled sensor node. The design and management of the nodes needs to meet the requirements of SDN and a major part of the design is that the node should be reconfigurable even after deployment via wireless communications. Examples of reconfigurable nodes presented for WSNs include a sensor node based on modularity [138] using the Field Programmable Gate Array (FPGA) as the node control unit [139] due to the re-configurable property of FPGA hardware unlike micro-controllers. This enables the remote reconfiguration of sensor hardware over wireless networks which is essential for SDN-based management of WSNs. To reduce the energy consumption of FPGAs, state of the art designs have been developed to operate in ultra low power modes [62]. Another implementation of sensor re-configurability is a node design with a soft core processor and the capacity to be reconfigured remotely via OTAP techniques post deployment based on the FPGA as the control unit [140]. The systems proposed in [139,140] use a micro-controller to re-configure the FPGA as a minimisation technique for power consumption.

Further efforts in proposing implementations of reconfigurable nodes (SDN-enabled nodes) in SDN-based WSNs have been made in recent years. TinySDN [69,118] uses an SDN-enabled node structured into three parts namely the TinySdnP which performs a flow table update upon receiving a flow setup resonse, TinyOS application which creates data packets and places them on the network and ActiveMessageC which is a component of TinyOS managing and providing a programming interface with radio hardware on the sensor. Miyazaki et al. [136] propose a SDWSN based on role generation and delivery to and from a reconfigurable node. The node makes use of a modular combination of FPGA and micro-controller for an energy efficient node that can be reprogrammed via OTAP to perform various roles. The micro-controller is used to alter the network behaviour while the FPGA handles heavier data processing tasks. Other enabling hardware such as computer servers for role or task generation and communication modules for the multi-hop wireless link have been presented for implementation in SDN-based management for WSNs [65,136].

## 6. Discussion

**Figure 5:**
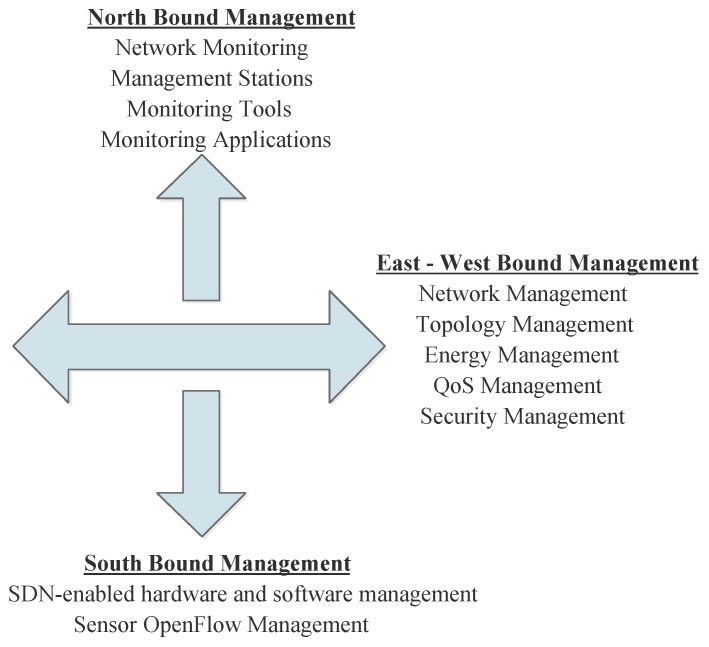
SDN-based management abstractions for WSNs.

In this review, several contributions to management of WSNs based on SDN have been made. It can generally be concluded that the overall SDN-based management of WSNs be looked at as being developed around the north bound, east-west bound and south bound abstractions of the SDN architecture. These are classified based on the location of the management control referred to as the manager. Figure 5 below illustrates this management view.

East-West bound management can further be looked at as central management, several proposals have been made towards improving management of control plane functions. Core to the function of SDN-based management of WSNs is the network configuration and topology management. Managing how the network operates efficiently and how nodes in the data plane communicate with the controller nodes and peripherals is the goal of developing a network configuration and topology management architecture. Soft-WSN an architecture based on East-West management has been proposed and tested by Bera et al. [9]. Soft-WSN is a software defined WSN management system for IoT providing an application-aware service consisting of an application, control and data layers. The architecture is based on centralized device (enabling hardware) and network configuration policies provisioned on the controller design. Experimental results on Soft-WSN show an enhanced energy efficiency, traffic overhead and data delivery ratio in a network [9].

To ensure SDN integration in WSNs, proposals such as Sensor OpenFlow, SDWN and SDN-WISE techniques which provide solutions to designing and managing WSNs based on SDN in terms of flow setup, in-network data processing and traffic management and general management of flow rules have been made. In managing the topology Smart, SDCSN and TinySDN provide an efficient high-level architecture resulting in efficient resource allocation. However, Smart does not provide for distributed controllers compared to SDCSN and TinySDN posing a greater risk in system security and reliability once the base station controller in Smart comes under attack. The network virtualisation demonstrated in the virtual overlay topology introduces greater flexibility in managing and monitoring networks. In managing localization of nodes, localization algorithms have been proposed to improve the accuracy of locating nodes. Zhu et al. [100] for example present a localization algorithm that does not only improve the performance of the localization but also conserve energy which is a vital component when designing and managing WSNs based on SDN. In tandem with management of node localization is mobility management which takes into account the movement of nodes into and out of the network. An attempt at managing mobility has been made by Zhou et al. [87] outlining the steps for handling node mobility in and out of a SDWSN however, there is room for research in SDN-based management of node mobility.

In terms on communication management, the importance of using multi-hop techniques in an energy constrained network has been emphasised. As data is carried and passed on from node to node, less energy is required and it also allows for data aggregation which reduces redundancy in the network. The challenge with this for enabling SDN-based management is the requirement for communication between the global controller and the nodes on the network as it may happen that a link may break once any of the nodes acting as repeaters falls out of the network. A more reliable communication scheme would integrate long range radio between each of the nodes and the controller although it may be argued that such a system would be less energy efficient. One solution would be investigating the possibility of using low power long range radio schemes such as LoRa [109]. In addition, to reduce rigidity in choice of design or communication scheme due to energy constraints for WSNs based on SDN, there is need for integration of energy harvesting mechanisms in the network. Methods to reduce the overhead on control data such as the proposed reinforcement learning technique [123] or a promising energy harvest method that uses wireless power transfer from a transmitting generator to the nodes in the field [126] can be further developed and investigated to reduce the energy bottleneck that exists in WSNs. In SDN-based energy management of WSNs, implementing a mix of proposed schemes to reduce and monitor energy consumed in the network such as duty cycling, data aggregation, smart [14] and multi-task methods [122] with energy harvesting techniques would result in higher energy efficiency of the network. It is also worthwhile to mention here that sensor node design is also focused on minimizing the energy consumed by use of low power devices in the electronic design while meeting the required QoS. In Table 5 various SDN-based management schemes and techniques for WSNs discussed in this paper are evaluated against the design criteria required for improved management.

## 7. Open Challenges

Generally, there is significant and on going contributions in investigating SDN-based management of networks, topologies, energy, QoS and enabling technologies for WSNs. However, a number of open challenges still exist:

### 7.1. East West Bound Management

With increase in sensor nodes to hundreds or thousands, use of a single controller becomes inefficient in terms of maintaining a global network view and data collection therefore proposals have been made towards using multiple controllers to solve this issue [28]. SDN-based management of communication protocols between distributed controllers in the control plane while maintaining a logically centralized and global view of the entire wireless sensor network remains an open challenge. This includes development of East-West bound APIs for efficient control of data flow in the data plane.

### 7.2. Network and Topology Management

Most resource management techniques available such as Smart and SDWN have the problem of not addressing overhead in control traffic which is costly for resource constrained WSNs. Therefore, further investigation on how to reduce in-band control traffic between the control and data planes is required. Efficient techniques for in-band communication of control and data traffic can be proposed and implemented.

Context based clustering can be studied further using other techniques other than HyperFlow to efficiently implement the logical controller concept while making use of its improved performance metrics such as improved scalability and reduced latency. Aspects of network decomposition hierarchies can be looked into to minimize control traffic and improve energy efficiency. Further investigation is also required to determine the number of controllers needed and their placement given an SDN-based WSN topology similar to the analysis done in [141] for WANs.

SDN-based network monitoring for WSNs is an essential part of network management that still remains largely unexplored. There is need for more quick and efficient network monitoring techniques for WSNs based on SDN. Node localisation is another issue that has the potential to be improved by SDN, conventional WSNs may require nodes to determine their geographical location based on information from other nodes [5] thus expending more energy. This task can be moved to the controller by virtue of its global position reducing the energy consumption on the nodes. In addition, novel localization techniques can be developed harnessing the potential of SDN.

### 7.3. Security Management

The existence of a logically centralized controller and cluster heads in the SDN-based architecture for WSNs calls for efficient management of security. This includes investigation and development of support for securing the controller in both distributed and centralized architectures in addition to counter measures whenever a cluster head or controller is attacked. There is also an open challenge in development of SDN-based key management systems and other encryption techniques for WSNs.

### 7.4. QoS and Mobility Management

Performance metrics such as latency, reachability and mobility remain open areas of research in SDN-based management of WSN QoS. There is need for more investigations in the controller placement problems to improve these performance metrics. Wang et al. [142] for example, have proposed a K-means algorithm to optimize controller placement thus minimizing network latency. Statistical analysis and artificial intelligence to predict fault occurrence in the network are also being investigated [24] for management of a robust SDN-based WSN. Investigation on the feasibility of mobility management techniques implemented in cellular networks being used in node mobility management of WSNs based on SDN is also a worthwhile area of research. Back up techniques upon node failure such as function alternation [65] can be investigated further based on leveraging SDN to enable efficient and simpler function alternation. To ensure QoS control and monitoring in the benchmarks and Key Performance Indicators(KPIs) need to integrated in the management framework developed.

### 7.5. Energy Management

Energy is a scarce resource in WSNs and constant research in this area is necessary to ensure efficient energy use in WSNs. A common technique to minimize the energy use is by control of sleep and wake states of sensor nodes however SDN-based management of WSNs requires a robust link between the control and data planes for effective management and thus should be investigated further to reduce unreliability in communication between the planes as a result. A paradigm shift is required to move away from data aggregation and further develop efficient long range low power radio techniques such as LoRa. Low power single hop communication techniques like this can also save energy by shifting in-network processing to the controller leaving the energy deprived sensor nodes with the task of only forwarding data to the controller and going back to sleep. Other improvements of energy efficiency based on reduction of overhead control traffic and redundancy in routing tasks are also a promising area of research. While new methods of extending network lifetime through wireless power transfer [126] have been proposed, the feasibility of implementing such a technique in a large-scale WSN based on SDN has to be addressed in terms of the scalability-cost ratio.

### 7.6. Enabling Technologies

Advances in microelectronic design and manufacture has allowed for miniaturisation of node hardware and at a lower production cost a factor which is useful in large-scale applications and allows for placement of redundant nodes in a network. However, there is need for continuous research in developing increased energy efficiency while keeping these nodes small and at a reduced cost. The energy harvest mechanisms is one such area of development, there is room for developing batteries that support quick and efficient charging at lower currents. A characteristic like this would be useful in harvesting solar energy for example. Integration of these nodes into the SDN architecture will also require further development of software and hardware to enable the SDN functionality and also provide support for multiple retasking, inter-plane data exchange via APIs and communication protocols (Flow-Sensor [128], FlowVisor [143], HyperFlow [93]. etc.). There is also need to develop some form of modularity in both software and hardware to allow for use in multiple applications and enable easy system maintenance. In terms of choice of using FPGA, micro-controller or a hybrid of both in SDN sensor node design, it can be argued that it vastly depends on energy efficiency, cost and nature of application. There are still possibilities of integrating low power FPGAs however the cost impact for application in large-scale networks also needs to be accounted for. Generally, novel hardware and software needs to be developed to improve support for the measurement, analysis and decision making process for IoT applications such as smart water grids while allowing compatibility with the integrated management framework.

### 7.7. WSN Management Framework

In the future, a development trend of more than just concepts and ideas of proposed management techniques is required; more work is needed for proof of concept and actual deployments to allow for a deeper understanding of the necessary architectures and tools for SDN-based management of WSNs. There have been attempts for actual deployment of proposed management mechanisms such as the case of SDN-WISE, the attempt by Miyazaki et al. [136] to build a reprogrammable WSN based on SDN and more recently Soft-WSN but there is still room for many such implementations. Areas such as distributed management based on distributed controllers, low power and long range SDN-based management of WSNs need investigation. As regards to network management, there is need for development of a WSN management framework leveraging SDN to ensure sensors/actuators are operating correctly and the heterogeneous SDN-based WSN is properly configured and managed. The management framework can be broken down to manage various aspects of the WSN and it should also be dynamic enough to be used for various applications. Just like MANNA and BOSS network management systems were presented for ordinary WSNs exploring how SDN can improve the performance of these NMSs would prove useful.

## 8. Conclusions

This paper reviewed the various contributions to managing WSNs and techniques available for SDN-based management of WSNs. The SDN paradigm has introduced flexibility and simplicity in managing wireless sensor networks, despite having different vendor specific hardware in the network. A highlight of the main real-world WSN applications and how SDN would improve the management of the applications was presented. However, the inherent properties of WSNs do not permit the easy integration of SDN as it was initially meant for traditional wired/wireless address-centric networks which are different from the data-centric WSNs. This paper also focused on the generic architecture of SDN-based WSNs and reviewed the management schemes available to ensure efficient functionality of the network. A review of the management classifications, namely management of the network configuration, topology, QoS, energy, security, network monitoring and enabling technologies with a further focus on SDN-enabled node hardware and software, was made. Furthermore, an attempt to define the overall management of WSNs based on SDN as abstractions of the north, south, east-west bound architecture of SDN had been presented. A discussion and summary was presented for the various proposals and work done necessary for management of WSNs based on SDN. However, there still exists a mix of open challenges available for effective SDN-based management of WSNs and they have been discussed in this paper with an emphasis on the need for actual implementations and test beds for effective evaluation of proposals and concepts. For a future leading to improved management of WSNs based on the Software Defined Networking paradigm; novel sdn-enabled sensor hardware, development of efficient SDN techniques for WSN implementation, novel contributions to SDN-based power management and improved performance assessment of SDN-based management architectures for WSNs are expected.

## Figures and Tables

**Table 1:** Available WSN management systems evaluation against the design criteria.

Management Scheme	Functionality	Energy Efficiency	Robustness	Scalability	Adaptability
MANNA [25]	Policy based framework, fault detection	NA	NA	NA	NA
BOSS [32]	Network state retrieval, power management	Yes	Yes	No	Yes
Agilla [31]	Event detection	Yes	No	No	Yes
Sectoral Sweeper [31]	Switching node on/off	Yes	No	No	No
Intelligent Agent- Based Power Management [31]	low power management	Yes	No	No	Yes
Mobile Agent Based Power Management [31]	Policy based management	Yes	Yes	No	Yes
RRP [31]	Data aggregation	Yes	No	No	No
SNMS [31]	Health and event data collection	Yes	Yes	No	No
SNMP [31]	Network function definition and monitoring	No	Yes	No	Yes
WSNManagement [33]	Performance and fault management	No	Yes	No	Yes
WinMS [31]	Synchronisation, local repair and state retrieval	Yes	Yes	Yes	Yes
SenOS [31]	Triggering node on/off	Yes	No	No	No
AppSleep [31]	Power Management	Yes	Yes	Yes	No
Energy level management [31]	Power management	Yes	Yes	No	No
EASA [44]	Self-sustaining energy management	Yes	Yes	No	No
MOTE-VIEW [52]	Network state and visualisation	Yes	No	Yes	No
EPMOSt [57]	Passive network monitoring	Yes	Yes	No	Yes

**Table 2:** SDN-based network and topology management architectures.

Management Architecture	Management Feature	Controller Configuration	Control Traffic Channel	Configuration and Monitoring	Scalability and Localization	Communication Management
Sensor OpenFlow [20,21]	SDN support protocol	Distributed and Centralized	in-band and out-band	√		√
SDWN [60]	Duty cycling, aggregation and routing	Distributed	in-band	√		
SDN-WISE [78]	programming simplicity, aggregation	Distributed	in-band	√		
Smart [14]	Efficiency in resource allocation	Centralized	in-band		√	
SDCSN [88]	Network reliability and QoS	Distributed	in-band		√	
TinySDN [69,118]	in-band traffic control	Distributed	in-band		√	
Virtual Overlay [59,87,90]	network flexibility	Distributed	in-band		√	
Context-based [91,92]	network scalability and performance	Distributed	in-band		√	
CRLB [100]	node localization	Centralized	in-band		√	
multi-hop [108]	traffic and energy control	Distributed and Centralized	in-band			√
TinySDM [117]	network task measurement	-	in-band	√		

**Table 3:** SDN-based Energy management schemes for WSNs.

Scheme	Features	Controller Architecture	Enabling Technology
SDWN [60]	Duty cycling, data aggregation	Distributed	Software, Hardware
Smart [14]	Resource allocation	Centralized	Software and Hardware
Multi-task [122]	Resource allocation, QoSen, scheduling	Centralized	Software
SDWSN-RL [123]	Load balancing, traffic control	Distributed	Software
Wireless power transfer [126]	Energy harvest, Optimization, efficiency	Centralized	Software, hardware

**Table 4:** Enabling node operating systems for SDN-based management of WSNs: A comparison.

OS	Language	Memory Management	Implementation for
TinyOS	NesC	Static	TinySDN [69,118], TinySDM [117], mobility [101]
Contiki	C	Dynamic	SDN-WISE [135]
MANTIS	C	Dynamic	-
Nano-RK	C	Static	-
LiteOS	Lite C++	Dynamic	-

**Table 5:** SDWSN management scheme evaluation against design criteria.

Management Scheme	Energy Efficiency	Robustness	Scalability	Adaptability
Sensor OpenFlow [20,21]	-	Yes	Yes	Yes
SDWN [60]	Yes	Yes	-	Yes
Smart [14]	Yes	No	No	Yes
SDN-WISE [78]	Yes	Yes	Yes	Yes
SDCSN [88]	Yes	Yes	Yes	Yes
TinySDN [69,118]	Yes	Yes	Yes	Yes
Virtual Overlay [59,87,90]	-	Yes	Yes	Yes
Multi-task [122]	Yes	-	-	Yes
SDWSN-RL [123]	Yes	Yes	Yes	Yes
Wireless power transfer [126]	Yes	Yes	Yes	-
Function alternation [65]	Yes	Yes	Yes	Yes
Statistical machine learning [24]	-	Yes	-	-
Context-based [91,92]	-	Yes	Yes	Yes
Soft-WSN [9]	Yes	Yes	-	Yes
